# Transient elastography as a screening tool for liver fibrosis in a large hemodialysis population

**DOI:** 10.1038/srep46458

**Published:** 2017-04-19

**Authors:** Ben-Chung Cheng, Yi-Hao Yen, Jung-Fu Chen, Cheng-Kun Wu, Kuo-Chin Chang, Po-Lin Tseng, Ming-Chao Tsai, Ming-Tsung Lin, Jung-Ting Lin, Jin-Bor Chen, Tsung-Hui Hu

**Affiliations:** 1 Division of Nephrology, Department of Internal Medicine, Kaohsiung Chang Gung Memorial Hospital and Chang Gung University College of Medicine, Kaohsiung, Taiwan; 2 Division of Hepato-Gastroenterology, Department of Internal Medicine, Kaohsiung Chang Gung Memorial Hospital and Chang Gung University College of Medicine, Kaohsiung, Taiwan; 3 Division of Endocrinology & Metabolism, Department of Internal Medicine, Kaohsiung Chang Gung Memorial Hospital and Chang Gung University College of Medicine, Kaohsiung, Taiwan

## Abstract

Metabolic syndrome, an etiological factor in non-alcoholic fatty liver disease (NAFLD), is often present in hemodialysis patients. Little is known about the prevalence of, and factors associated with, liver fibrosis in hemodialysis populations. We used transient elastography (TE) to investigate these phenomena. 659 patients treated with chronic hemodialysis were enrolled. We excluded those with excess alcohol intake, liver stiffness measurement (LSM) failure, or unreliable LSM values. LSM ≥8.0 kPa was used as a cutoff suggesting clinically relevant fibrosis. Controlled attenuation parameter (CAP) ≥ 232.5 dB/m was used as a cutoff suggesting steatosis. 333 patients (50.5%) had steatosis, 159 (24.1%) had hepatitis B or C, and 149 (22.6%) had LSM ≥8.0 kPa. In multivariable analyses, male gender (OR: 2.16; 95% CI: 1.29–3.63; P = 0.004), overweight body habitus (OR:2.31; 95% CI: 1.35–3.94; P = 0.002), high AST level (OR:1.08; 95% CI: 1.04–1.12; P < 0.001), low albumin level (OR: 0.25; 95% CI: 0.12–0.53; P < 0.001), low creatinine level (OR: 0.89; 95% CI: 0.79–1.00; P = 0.05) and low platelet count (OR: 0.99; 95% CI: 0.99–1.00; P < 0.001) were associated with LSM ≥8.0 kPa. We thus conclude that hemodialysis patients have a high prevalence of NAFLD and clinically relevant fibrosis. NAFLD may be an important determinant of clinically relevant fibrosis in hemodialysis populations.

Non-alcoholic fatty liver disease (NAFLD) is the most common chronic liver disease, affecting 15–40% of the population worldwide^1^. Around 20–30% of patients with NAFLD have nonalcoholic steatohepatitis (NASH), which will eventually progress to cirrhosis and hepatocellular carcinoma in 10–20% of patients^2^,^3^,^4^. NAFLD is strongly associated with the components of metabolic syndrome (central obesity, hypertension, hyperglycemia, and dyslipidemia) and is now regarded as the liver manifestation of metabolic syndrome^5^,^6^,^7^.

Moreover, the main risk factors responsible for the development of NAFLD (which are also the components of metabolic syndrome) are commonly observed in dialysis patients. Therefore, it is logical to expect that end-stage renal disease (ESRD) patients maintained on hemodialysis would also have a high prevalence of NAFLD.

Transient elastrography (TE) is a non-invasive test of liver fibrosis that is quick and easy to perform and causes no discomfort. It has high accuracy and reproducibility when used to detect advanced fibrosis and cirrhosis. The controlled attenuation parameter (CAP) is a measurement of the degree of ultrasound attenuation caused by hepatic fat at the central frequency of the FibroScan^8^. CAP measurements have been shown to be accurate in estimating the amount of liver fat^9^,^10^,^11^. It is thus now possible, using the non-invasive technique of TE, to measure liver fat and fibrosis simultaneously.

To date, only a limited number of studies have been performed focusing on the prevalence and risk factors for liver fibrosis in ESRD patients. Due to the fact that alanine aminotransferase (ALT) tends to be within the normal range in ESRD patients, we used one of the best available non-invasive tests (that is, TE) to evaluate liver fibrosis in this special population. The aim of our study was to investigate the prevalence of, and factors associated with, clinically relevant liver fibrosis, as measured by TE, in a large cohort of ESRD patients on maintained hemodialysis.

## Methods

### Patients

Patients treated at our center (Kaoshiung Chang Gung Memorial Hospital) for chronic hemodialysis between April and September of 2014 were considered for inclusion in our study. These patients received hemodialysis for at least 6 months. We excluded those patients with excess alcohol intake, meaning consumption of >21 alcoholic drinks per week in men and >14 alcoholic drinks per week in women over the preceding 2 years^12^, and also excluded others for technical reasons, including failure of the FibroScan or unreliable liver stiffness measurement (LSM) (i.e., interquantile range (IQR) > 0.3). The study protocol adhered to the ethical guidelines of the 1975 Declaration of Helsinki and was approved by the ethical committee of Chang Gung Memorial Hospital. Written informed consent was obtained from each of the participants in this study.

### FibroScan examination

LSM and CAP results were obtained using a FibroScan device (Echosens, Paris, France). All the patients fasted for at least 2 h before the exam and all the exams were performed before dialysis. The LSM was represented by the median of 10 measurements and was considered reliable only if at least 10 successful acquisitions were obtained and the IQR-to-median ratio of the 10 acquisitions was ≤0.3. The CAP was represented by the median value. CAP measurements were considered reliable if 10 successful acquisitions were obtained. The M probe was used for all patients. Two operators who each had >6 years of experience with the FibroScan device and had performed >3000 procedures prior to the study performed the procedures. LSM ≥8.0 kPa and >13.0 kPa were taken as cutoffs suggesting clinically relevant liver fibrosis and cirrhosis, respectively^13^,^14^,^15^. CAP ≥ 232.5 dB/m was taken as the cutoff level suggesting hepatic steatosis^16^.

### Fibrosis 4 calculator (FIB-4) as a comparator for fibrosis assessment

We used FIB-4 as a comparator for liver fibrosis assessment. FIB-4 is an accepted, non-invasive procedure for the identification of cases at risk of advanced fibrosis as recommended by the European Association for the Study of the Liver (EASL) guideline^17^. FIB-4 ≥2.67 was defined as advanced fibrosis in NAFLD patients^18^.

### Biological parameters

The same day as the TE measurements were conducted, the following clinical parameters were recorded: age, gender, body mass index (BMI), diabetes mellitus (DM) status, fasting lipid profile, fasting sugar level, hepatitis B surface antigen (HBsAg), hepatitis C virus antibody (anti-HCV), liver function tests, complete blood count, and creatinine. Biological parameters were measured before the hemodialysis.

### Statistical analysis

The baseline characteristics and clinical variables were summarized as mean ± standard deviation, median (25th–75th percentile), or percentage, and the Student’s t test, chi-square test, or Fisher’s exact test were used as appropriate to assess the significance of differences in the distributions of the characteristics and variables. Multivariate logistical regression analyses were conducted to identify patient characteristics independently associated with clinically relevant fibrosis. A univariate analysis was first performed on each of the considered independent variables to select candidate variables for the multivariate analyses. The performance of the LSM for diagnosing clinically relevant fibrosis compared with that of FIB-4 was assessed using the area under the receiver operating characteristic curve (AUROC). In all analyses, a p-value < 0.05 was considered statistically significant. Statistical analyses were performed using Stata software version 11.0.

## Results

### Study population

Six hundred and fifty-nine patients were enrolled in this study. The baseline characteristics of the patients are shown in Table 1: 51.8% of the patients were women, the mean age of the patients was 61.9 ± 11.8 years, and the mean BMI of the patients was 22.3 ± 3.4 kg/m2. In addition, the median LSM was 5.9 kPa, 50.5% of the patients had hepatic steatosis, and 159 (24.1%) had positive viral serology (72 were HBsAg positive and 95 were anti-HCV positive, while 8 were both HBsAg and anti-HCV positive).

### Factors associated with clinically relevant fibrosis

**Figure 1:**
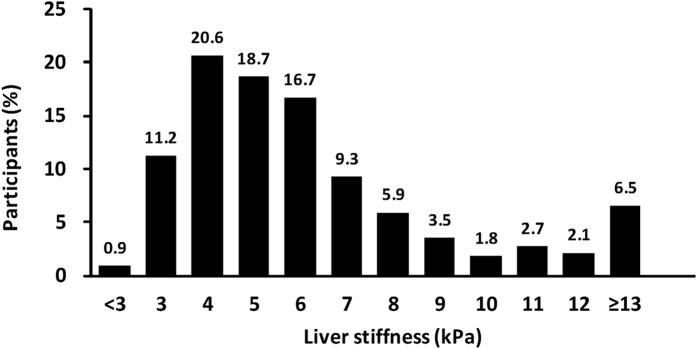
Distribution of liver stiffness measurement values in our cohort of all patients.

LSM ≥8.0 kPa, suggesting the presence of clinically relevant fibrosis, was detected in 149 participants (22.6%). The distribution of LSM values in our cohort of 659 patients is illustrated in Fig. 1. Multivariate analyses showed that male gender (odds ratio [OR]: 2.16, 95% confidence interval [CI]: (1.29–3.63), P = 0.004), overweight body habitus (i.e., 24 ≤ BMI < 30 (OR: 2.31, 95% CI: 1.35–3.94, P = 0.002)), high AST level (OR: 1.08, 95% CI: 1.04–1.12, P < 0.001), low albumin level (OR: 0.25, 95% CI: 0.12–0.53, P < 0.001), low creatinine level (OR: 0.89. 95% CI: 0.79–1.00, P = 0.05) and low platelet count (OR: 0.99. 95% CI: 0.99–1.00, P < 0.001) were independent factors associated with LSM ≥8.0 kPa (Table 2).

### Diagnostic performance of the LSM for FIB-4-identified clinically relevant fibrosis

Using FIB-4 ≥ 2.67 as a surrogate gold standard for clinically relevant fibrosis, the diagnostic performance of LSM ≥8.0 kPa in all patients was as follows: AUROC = 0.807, 95% CI = 0.64–0.97, sensitivity = 83.33%, specificity = 78.09%, positive predictive value (PPV) = 3.29%, and negative predictive value (NPV) = 99.81%. The diagnostic performance of LSM ≥8.0 kPa in patients without chronic hepatitis B or C was as follows: AUROC = 0.821, 95% CI = 0.66–0.99, sensitivity = 83.33%, specificity = 80.87%, PPV = 4.90%, and NPV = 99.76%.

### Factors associated with liver cirrhosis

Forty-three (6.5%) of the 659 cases were found to have LSM >13.0 kPa. Of those patients, 16 (37.2%) were female, the mean age was 64.6 ± 9.7 years, 13 (30.2%) were overweight (BMI 24–30), 2 (4.7%) were obese (BMI > 30), 11 (25.6%) had hepatic steatosis, 18 (41.9%) had DM, and 18 (41.9%) were HBsAg and/or anti-HCV positive. Multivariate analyses showed that male gender (OR: 2.75, 95% CI: 1.16–6.52, P = 0.02), obesity (i.e., BMI ≥30 (OR: 13.64, 95% CI: 2.06–90.50, P = 0.007)), high AST level (OR: 1.06, 95% CI: 1.02–1.11, P = 0.007), low creatinine level (OR: 0.76, 95% CI: 0.62–0.94, P = 0.01) and low platelet count (OR: 0.99, 95% CI: 0.98–0.99, P < 0.001) were independent factors associated with LSM >13.0 kPa.

### Factors associated with steatosis

Multivariate analyses showed that old age (OR:1.03, 95% CI: 1.01–1.05, P < 0.001), overweight body habitus (i.e., 24 ≤ BMI < 30 (OR: 2.12, 95% CI: 1.35–3.33, P = 0.001)), DM (OR: 2.58, 95% CI: 1.70–3.92, P < 0.001), high creatinine level (OR: 1.18, 95% CI: 1.06–1.30, P = 0.002), HDL–C <40 mg/dL (for males) or <50 mg/dL (for females) (OR: 1.82, 95% CI: 1.21–2.73, P = 0.004), and triglyceride >150 (OR: 3.46, 95% CI: 2.13–5.61, P < 0.001) were independent factors associated with steatosis (Table 3).

### Factors associated with clinically relevant fibrosis in patients with NAFLD

A subgroup analysis was performed to investigate the factors associated with LSM in patients with NAFLD. Using CAP ≥232.5 dB/m as the definition of hepatic steatosis and excluding patients who were HBsAg or anti-HCV positive, 248 patients were diagnosed with NAFLD. Among those patients, the mean age was 60.8 ± 10.9 years, the mean BMI was 23.9 ± 3.5 kg/m2, the median LSM was 5.9 kPa (4.7–7.5), and 50 (20.2%) patients had LSM ≥8.0 kPa. Multivariate analyses showed that overweight body habitus (i.e., 24 ≤ BMI < 30 (OR = 4.13, 95% CI: 1.69–10.10, P = 0.002)), obesity (i.e., BMI ≥30 (OR = 9.44. 95% CI: 1.80–49.49, P = 0.008)), high AST level (OR = 1.21, 95% CI: 1.11–1.31, P < 0.001), and low ALT level (OR = 0.93. 95% CI: 0.87–0.99, P = 0.02) were independent factors associated with LSM ≥8.0 kPa in NAFLD patients (Table 4).

## Discussion

The prevalence of NAFLD in patients undergoing dialysis remains unknown.

The main risk factors responsible for the development of NAFLD (which are also the components of metabolic syndrome), however, are commonly observed in dialysis patients. Therefore, it is logical to expect that ESRD patients would also have a high prevalence of NAFLD. In this study, the prevalence of NAFLD in ESRD patients was 50.5%, which is higher than the incidence in the general population (15–40%)^1^.

Serologic testing has clearly demonstrated that HCV infection is highly prevalent among ESRD patients and is a serious cause of increased morbidity and mortality in this group. In 2002, the prevalence of HCV infection across dialysis centers in the United States was approximately 8%^19^,^20^. In some European dialysis centers, the yearly incidence of HCV infection reportedly ranges from 0.4 to 16.0%^21^. The prevalence of HCV infection in this study was 14.4%, which is comparable to the rates reported in previous studies. Since Taiwan is an endemic country for HBV infection, the prevalence of HBV in Taiwan is 17.3%^22^, and in this study, the prevalence of HBV infection was found to be 10.9%. For the above reasons, ESRD patients have a higher prevalence of chronic liver disease. Therefore, it is logical to survey liver disease in this population. To the best of our knowledge, this is the first large-scale study to use TE to survey liver disease in an ESRD population. In this large hospital cohort, 22.6% of the ESRD patients had LSM ≥8.0 kPa. Male patients, overweight patients, and patients with higher AST levels and lower albumin, creatinine, and platelet levels were more likely to have LSM ≥8.0 kPa.

Koehler *et al*. reported that 5.6% of the participants in their large Caucasian population-based study of older adults had LSM ≥8.0 kPa. Old age, high ALT level, smoking, HBsAg, or anti-HCV positivity and the combined presence of DM and steatosis were associated with LSM ≥8.0 kPa in their multivariable analyses^23^. Roulot *et al*. reported that 7.5% of participants had LSM > 8 kPa in their large Caucasian population-based study of older adults. Age ≥ 57 years, BMI ≥ 30 kg/m^2^, elevated waist circumference, DM, gamma-glutamyl transpeptidase (r-GT) ≥ 45 IU/L, and ALT ≥ 40 IU/L were associated with LSM >8 kPa in their multivariate analyses^14^. The prevalence of clinically relevant fibrosis (LSM ≥8.0 kPa) was higher in our study compared with previous studies that enrolled older adult populations. This was possibly due to the higher prevalence of viral hepatitis B or C in our study (24%), whereas the prevalence was 0.8% in the Koehler *et al*.^23^ study and less than 1% in the Roulot *et al*.^14^ study. Using CAP ≥ 232.5 dB/m as the definition of steatosis, we found that 50.5% of the cases in our study had steatosis. In contrast, using ultrasound to diagnose steatosis, Koehler *et al*. found that only 35.5% the cases in their study had steatosis^23^. Therefore, the higher proportions of viral hepatitis and NAFLD in our study could explain the higher proportion of clinically relevant fibrosis in this study. Notably, BMI and aminotransferase were the same independent factors associated with LSM ≥8.0 kPa in both our study and previous studies^14^,^23^.

Although TE is easy to perform, it is unlikely that clinicians can apply it to all hemodialysis patients because of the large number of such patients. Therefore, it is important to identify those patients who are at risk of advanced liver disease. To that end, male gender, higher BMI, higher AST level, and lower albumin, creatinine, and platelet levels were the independent factors found to be associated with increased LSM in this study. As such, patients with these risk factors may benefit from TE assessment.

In this study, 43 (6.5%) of the 659 cases were found to have LSM >13.0 kPa. Of those patients, 27 were male, 15 were overweight or obese, 11 had steatosis, 18 had DM, and 18 were HBsAg and/or anti-HCV positive. In contrast, Koehler *et al*. reported that 19 (0.6%) of the participants in their population-based study of older adults had LSM >13.0 kPa. Among those patients, most were female, the mean BMI was 28.7 kg/m2, two participants had excess alcohol intake, 11 participants had steatosis, and 7 had DM^23^. Obesity, DM, and steatosis were risk factors for liver cirrhosis in both our study and in the Koehler *et al*. study^23^.

Two hundred and forty-eight cases were diagnosed with NAFLD in our study, and 20.2% of those cases had LSM ≥8.0 kpa. Multivariate analyses showed that BMI, AST and ALT were independent factors associated with LSM ≥8.0 kpa, and these factors were also the independent factors associated with LSM ≥8.0 kpa in the entire population in this study. These findings suggest that NAFLD may be an important determinant of clinically relevant fibrosis in hemodialysis populations. Since NAFLD has become the predominant cause of chronic liver disease in many parts of the world^24^, NAFLD will become a more urgent health issue in hemodialysis populations.

Fibrosis is the most important prognostic factor in NAFLD and is correlated with liver-related outcomes and mortality^25^. The use of the NAFLD fibrosis score (NFS) is suggested in patients with suspected NAFLD, as recommended by both the American Association for the Study of Liver Diseases (AASLD) and EASL guidelines^12^,^17^. A previous study used the combination of LSM with NFS, is able to accurately diagnose the presence of severe liver fibrosis^26^. Severe fibrosis was defined as fibrosis  ≥ F3 according to the NASH Clinical Research Network system^27^. Patients with severe liver fibrosis should be screened with endoscopy for esophageal varices and ultrasonography for hepatocellular carcinoma^28^. According to these studies, we could use the combination of LSM with NFS to diagnose the presence of severe liver fibrosis. Patients with severe liver fibrosis should be screened with endoscopy for esophageal varices and ultrasonography for hepatocellular carcinoma.

Our study has the strength of a large sample size and the use of one of the best and most widely available non-invasive tests of liver steatosis and fibrosis; this is the first large-scale study to have used TE to survey liver disease in an ESRD population. However, our study also has some limitations. Liver biopsy is the gold standard to evaluate liver fibrosis. The cut-off value of LSM for diagnosing clinically relevant fibrosis in this study was obtained from non-uremic populations^13^,^14^,^15^. However, studies with liver biopsy and TE performed in hemodialysis patients are scarce, and all of the previous studies of this type involved HCV-infected patients^29^,^30^,^31^,^32^,^33^,^34^,^35^. Therefore, we used FIB-4 as a comparator test for clinically relevant fibrosis, and we found that the diagnostic performance of the LSM for FIB-4-identified clinically relevant fibrosis is relatively good (AUROC > 0.8).

In conclusion, hemodialysis patients have a high prevalence of NAFLD and clinically relevant liver fibrosis. Hemodialysis patients who are male, obese, and have higher AST levels and lower albumin, creatinine, and platelet levels are at particularly high risk and may be good targets for TE assessment. These findings suggest that NAFLD may be an important determinant of clinically relevant fibrosis in hemodialysis populations.

## Additional Information

**How to cite this article:** Cheng, B.-C. *et al*. Transient elastography as a screening tool for liver fibrosis in a large hemodialysis population. *Sci. Rep.* **7**, 46458; doi: 10.1038/srep46458 (2017).

**Publisher's note:** Springer Nature remains neutral with regard to jurisdictional claims in published maps and institutional affiliations.

## Figures and Tables

**Table 1:** Baseline characteristics of all patients.

Characteristic	Total (N = 659)
Age, years	61.9 ± 11.8
Female	51.8
BMI (kg/m2)	22.3 ± 3.4
Normal; BMI <24 (kg/m2)	71.6
Overweight; 24 ≤ BMI < 30 (kg/m2)	26.1
Obese; BMI ≥30 (kg/m2)	2.3
Presence of HBV or HCV (%)	24.0
DM (%)	32.8
AST (IU/L)	17 (14–22)
ALT (IU/L)	13 (10–19)
Bilirubin (mg/dL)	0.3 (0.2–0.4)
Albumin (mg/dL)	4.0 (3.7–4.2)
Creatinine (mg/dL)	10.7 (912–12.2)
Platelet (10^9^/L)	180 (144–216)
Stiffness (kPa)	5.9 (4.6–7.8)
HDL–C: <40 mg/dL(male) or <50 mg/dL(female)	48.3
LDL (mg/dL)	85.9 ± 30.6
Triglyceride > 150 (mg/dL)	34.9
Total cholesterol (mg/dL)	161.8 ± 38.0
Steatosis	
No Steatosis (CAP <232.5) (dB/m)	49.47
Steatosis grade 1 (232.5 ≥CAP <255) (dB/m)	20.49
Steatosis grade 2 (255 ≥ CAP <290) (dB/m)	17.00
Steatosis grade 3 (≥290) (dB/m)	13.05

Data are represented as mean ± standard deviation, median (25th–75th percentile), or percentage. BMI, body mass index; DM, diabetes mellitus; ALT, alanine aminotransferase; AST, aspartate aminotransferase; HDL, High-density lipoprotein; LDL, Low-density lipoprotein; CAP, Controlled Attenuation Parameter; HBV, hepatitis B virus; HCV, hepatitis C virus.

**Table 2:** Factors associated with liver stiffness measurement ≥8.0 kPa in univariate and multivariate analyses.

Variables	Univariate analysis	*P* Value	Multivriate analysis	*P* Value
	OR (95% CI)		OR (95% CI)	
Age, years	1.03 (1.01–1.05)	<0.001	1.01 (0.99–1.03)	0.48
Male	1.64 (1.13–2.37)	0.009	2.16 (1.29–3.63)	0.004
BMI				
Normal; BMI <24 (kg/m2)	1.00 (reference)		1.00 (reference)	
Overweight; 24 ≤ BMI < 30 (kg/m2)	1.43 (0.96–2.15)	0.079	2.31 (1.35–3.94)	0.002
Obese; BMI ≥30 (kg/m2)	1.39 (0.43–4.45)	0.582	3.67 (0.97–13.96)	0.06
Presence of HBV or HCV (%)	1.80 (1.21–2.69)	0.004	1.53 (0.95–2.47)	0.08
DM (%)	1.13 (0.77–1.66)	0.531	0.63 (0.38–1.03)	0.06
AST (IU/L)	1.08 (1.05–1.10)	<0.001	1.08 (1.04–1.12)	<0.001
ALT (IU/L)	1.03 (1.01–1.04)	<0.001	0.99 (0.96–1.01)	0.27
Bilirubin (mg/dL)	1.16 (0.81–1.67)	0.418	1.14 (0.75–1.72)	0.54
Albumin (mg/dL)	0.16 (0.09–0.29)	<0.001	0.25 (0.12–0.53)	<0.001
Creatinine (mg/dL)	0.89 (0.82–0.96)	0.002	0.89 (0.79–1.00)	0.05
Platelet (10^9^/L)	0.99 (0.99–0.99)	<0.001	0.99 (0.99–1.00)	<0.001
HDL-C: <40 mg/dL(male) or <50 mg/dL(female)	1.47 (1.02–2.12)	0.039	1.52 (0.91–2.55)	0.11
LDL (mg/dL)	0.99 (0.98–1.00)	0.002	1.00 (0.99–1.02)	0.76
Triglyceride >150 (mg/dL)	0.79 (0.53–1.17)	0.241	0.95 (0.52–1.74)	0.88
Total cholesterol (mg/dL)	0.99 (0.98–0.99)	<0.001	1.00 (0.99–1.01)	0.61
Steatosis				
No Steatosis (CAP <232.5) (dB/m)	1.00 (reference)		1.00 (reference)	
Steatosis grade 1 (232.5 ≥CAP <255) (dB/m)	0.72 (0.43–1.21)	0.215	0.80 (0.43–1.48)	0.47
Steatosis grade 2 (255 ≥ CAP <290) (dB/m)	0.91 (0.54–1.53)	0.731	1.07 (0.57–2.04)	0.83
Steatosis grade3 (≥290) (dB/m)	1.45 (0.86–2.46)	0.167	1.79 (0.88–3.62)	0.11

BMI, body mass index; DM, diabetes mellitus; ALT, alanine aminotransferase; AST, aspartate aminotransferase; HDL, High-density lipoprotein; LDL, Low-density lipoprotein; CAP, Controlled Attenuation Parameter; HBV, hepatitis B virus; HCV, hepatitis C virus.

**Table 3:** Factors associated with steatosis (CAP ≥ 232.5 dB/m) in univariate and multivariate analyses.

Variables	Univariate analysis	*P* Value	Multivriate analysis	*P* Value
	OR (95% CI)		OR (95% CI)	
Age, years	1.01 (1.00–1.03)	0.065	1.03 (1.01–1.05)	<0.001
Male	1.11 (0.82–1.51)	0.502	0.67 (0.43–1.04)	0.07
BMI				
Normal; BMI <24 (kg/m2)	1.00 (reference)		1.00 (reference)	
Overweight; 24 ≤ BMI < 30 (kg/m2)	4.24 (2.88–6.26)	<0.001	2.12 (1.35–3.33)	0.001
Obese; BMI ≥30 (kg/m2)	9.48 (2.12–42.48)	0.003	4.82 (0.98–23.79)	0.05
DM (%)	2.86 (2.03–4.02)	<0.001	2.58 (1.70–3.92)	<0.001
AST (IU/L)	0.98 (0.96–1.00)	0.021	0.99 (0.96–1.02)	0.40
ALT (IU/L)	0.99 (0.98–1.01)	0.258	1.00 (0.98–1.02)	0.65
Bilirubin (mg/dL)	0.22 (0.08–0.59)	0.003	0.68 (0.34–1.35)	0.27
Albumin (mg/dL)	1.44 (0.91–2.27)	0.121	1.12 (0.61–2.06)	0.71
Creatinine (mg/dL)	1.11 (1.04–1.19)	0.001	1.18 (1.06–1.30)	0.002
Platelet (10^9^/L)	1.00 (1.00–1.01)	0.001	1.00 (1.00–1.00)	0.43
HDL-C: <40 mg/dL(male) or <50 mg/dL(female)	3.74 (2.71–5.16)	<0.001	1.82 (1.21–2.73)	0.004
LDL (mg/dL)	1.00 (1.00–1.01)	0.462	1.00 (0.99–1.01)	0.47
Triglyceride >150 (mg/dL)	5.10 (3.57–7.30)	<0.001	3.46 (2.13–5.61)	<0.001
Total cholesterol (mg/dL)	1.00 (1.00–1.01)	0.092	1.00 (0.99–1.01)	0.41

BMI, body mass index; DM, diabetes mellitus; ALT, alanine aminotransferase; AST, aspartate aminotransferase; HDL, High-density lipoprotein; LDL, Low-density lipoprotein; CAP, Controlled Attenuation Parameter; HBV, hepatitis B virus; HCV, hepatitis C virus.

**Table 4:** Factors associated with liver stiffness measurement ≥ 8.0 kPa in univariate and multivariate analyses in patients with non-alcoholic fatty liver disease.

Variables	Univariate analysis	*P* Value	Multivriate analysis	*P* Value
	OR (95% CI)		OR (95% CI)	
Age, years	1.03 (1.00–1.06)	0.078	1.02 (0.97–1.06)	0.47
Male	1.15 (0.62–2.14)	0.657	1.33 (0.55–3.20)	0.53
BMI				
Normal; BMI <24 (kg/m2)	1.00 (reference)		1.00 (reference)	
Overweight; 24 ≤ BMI < 30 (kg/m2)	2.49 (1.29–4.81)	0.006	4.13 (1.69–10.10)	0.002
Obese; BMI ≥30 (kg/m2)	3.18 (0.87–11.61)	0.079	9.44 (1.80–49.49)	0.008
DM (%)	1.00 (0.54–1.88)	0.992	0.64 (0.28–1.42)	0.27
AST (IU/L)	1.11 (1.06–1.17)	<0.001	1.21 (1.11–1.31)	<0.001
ALT (IU/L)	1.02 (0.99–1.05)	0.218	0.93 (0.87–0.99)	0.02
Bilirubin (mg/dL)	3.51 (0.45–27.23)	0.230	8.02 (0.46–139.24)	0.15
Albumin (mg/dL)	0.43 (0.17–1.11)	0.080	0.48 (0.13–1.78)	0.27
Creatinine (mg/dL)	0.95 (0.84–1.08)	0.407	0.98 (0.80–1.20)	0.85
Platelet (10^9^/L)	1.00 (0.99–1.00)	0.097	1.00 (0.99–1.00)	0.18
HDL-C: <40 mg/dL(male) or <50 mg/dL(female)	2.03 (0.98–4.20)	0.058	1.83 (0.69–4.85)	0.22
LDL (mg/dL)	0.99 (0.98–1.00)	0.034	0.99 (0.96–1.01)	0.21
Triglyceride >150 (mg/dL)	1.51 (0.80–2.87)	0.207	1.20 (0.42–3.43)	0.74
Total cholesterol (mg/dL)	0.99 (0.98–1.00)	0.093	1.00 (0.98–1.03)	0.64
Steatosis				
Steatosis grade 1 (232.5 ≥CAP <255) (dB/m)	1.00 (reference)		1.00 (reference)	
Steatosis grade 2 (255 ≥ CAP <290) (dB/m)	2.26 (0.96–5.32)	0.062	2.20 (0.80–6.03)	0.13
Steatosis grade 3 (≥290) (dB/m)	3.64 (1.55–8.55)	0.003	2.94 (1.00–8.63)	0.05

BMI, body mass index; DM, diabetes mellitus; ALT, alanine aminotransferase; AST, aspartate aminotransferase; HDL, High-density lipoprotein; LDL, Low-density lipoprotein; CAP, Controlled Attenuation Parameter; HBV, hepatitis B virus; HCV, hepatitis C virus.
